# Community environmental assessment for migrant seniors’ mental health: a multi-attribute decision-making model

**DOI:** 10.3389/fmed.2026.1753627

**Published:** 2026-01-23

**Authors:** Zeyu Wu, Xiaopan Qi, Jialin Qin, Beini Cai, Shuo Kuang

**Affiliations:** 1 Faculty of Humanities and Arts, Macau University of Science and Technology, Taipa, Macao SAR, China; 2 School of Design and Creative Arts, Loughborough University, Loughborough, United Kingdom

**Keywords:** cause–effect relationship, community environment, DANP-V mode, elderly migrants, mental health

## Abstract

**Introduction:**

Elderly migrants face significant mental health risks and social isolation within the context of global aging and mobility. As their daily activities are highly community-centric, the residential environment is a critical determinant of their well-being; however, a systematic assessment framework tailored to this population is lacking. This study aims to address this gap by identifying key community environmental factors influencing their mental health and employing the DANP-V model to construct a systemic methodology that elucidates complex inter-factor causalities and establishes prioritized improvement strategies.

**Methods:**

We established a framework of community environmental factors based on the daily behaviors of elderly migrants. The Fuzzy Delphi Method (FDM) was used to screen and finalize 16 key indicators across six dimensions. The DANP-V model, a hybrid multi-criteria decision-making (MCDM) technique integrating DEMATEL, ANP, and VIKOR methods, was then applied to analyze the interrelationships and weights of these factors. The model was empirically tested through a case study in Qianshan Community, Zhuhai, China, using survey data from both domain experts (*n*=10) and elderly migrants (*n*=140).

**Results:**

The DANP-V analysis revealed a total performance gap of 0.495 for the case community, indicating substantial room for improvement. “Environmental Exposure” (D6, gap = 0.629) and “Self-Actualization” (D4, gap = 0.617) were the most deficient dimensions. Key criteria with the largest gaps included “Pet-Friendly Facilities” (C54, gap = 0.779), “Ambient Temperature” (C63, gap = 0.726), and “Ancestral Worship Sites” (C51, gap = 0.713). The Influential Network Relation Map (INRM) illustrated that factors like “Transportation & Mobility” (C53) and “Living Convenience Facilities” (C52) were influential predecessors, affecting other criteria.

**Discussion:**

The DANP-V model provides a systemic approach to assess and improve community environments for elderly migrants’ mental health, moving beyond isolated factors to address root causes within an interconnected system. The case study demonstrates that critical gaps often lie in culturally-specific (e.g., ancestral worship) and emotion-supporting (e.g., pet-friendly) elements, which are frequently overlooked. The study offers a robust framework for policymakers and designers to develop targeted, effective community improvement strategies.

## 1 Introduction

### 1.1 Research background

The 21st century has witnessed unprecedented global population migration. Economic reforms and urbanization have led to a large influx of internal migrants, with China’s migrant population experiencing explosive growth between 2010 and 2020 (1). This migration has primarily occurred from remote rural areas to densely populated urban areas and from impoverished inland regions to economically developed eastern coastal cities (2). In China, the migrant population—defined as individuals who leave their original place of household registration to settle in other regions (2)—is shaped by the household registration system (3, 4). Influenced by traditional Chinese family values, many older adults migrate with their children after retirement, assuming intergenerational care responsibilities. Driven by both population aging and the demand for intergenerational care, the number of elderly migrants in China continues to rise (5). Although elderly migration is largely voluntary, language and cultural differences, together with varying local policies, mean that elderly migrants face numerous pressures in their new environments, including fulfilling family responsibilities, understanding the social environment, and maintaining physical functioning (6). This pressure results in consistently higher rates of depression among elderly migrants compared with other older adult groups, and is also associated with a higher probability of developing age-related conditions such as stroke, insomnia, and dementia (7, 8). The social pressures brought about by migration have become a significant threat to the physical and mental health and overall quality of life of elderly migrants (9).

Given their age-related conditions, elderly immigrants experience mobility limitations due to health and environmental factors. Consequently, their daily activities are primarily centered around the community and are strongly influenced by the community environment (10). Community-based daily routines and activities facilitate social interaction among the elderly in a continuous and subtle manner, making such interactions more readily accepted (11). According to environmental design theory (12), the physical environment significantly influences social behavior. Previous studies have confirmed a link between the mental health of immigrants and their residential communities (13). Both the physical and social environments of a community—such as social support, trust, and cohesion—play important roles in shaping immigrants’ mental health (14). For example, walkability, accessibility, and green space are environmental factors that can positively affect residents’ mental health (15, 16). Furthermore, research on the availability of community recreation, healthcare, shopping, and services indicates that greater availability of such facilities and services is associated with better mental health outcomes (17). Community squares, activity centers, and public green spaces serve as key venues for social interaction among elderly immigrants. These spaces can effectively mitigate the negative effects of stress on physical and mental health through leisure activities (18), social engagement (19), and increased frequency of outdoor excursions (20).

### 1.2 Research objectives and questions

Research on the relationship between the community environment and mental health has been a focus of scholarly attention; however, previous studies have largely been conducted in Western contexts (5) and have primarily focused on indigenous populations (21). Consequently, the stress experienced by older migrants remains relatively underexplored (22–24). This study aims to systematically explore the community environmental factors influencing the mental health of elderly immigrants and propose improvement strategies. The research objectives are as follows: First, based on literature review, key community environmental factors affecting the mental health of elderly immigrants will be identified, and a community environment assessment scale will be constructed. Second, the correlations between various environmental factors will be analyzed, the influence weights of the assessment criteria will be established, and an influence network relationship diagram (INRM) will be constructed. Finally, an empirical case study will be conducted in an immigrant community in Zhuhai, China, and community environment improvement strategies will be developed based on the assessment scale. To achieve these objectives, the study will answer the following questions:

RQ1: Which community environmental factors have a key impact on the mental health of elderly immigrants?

RQ2: What are the interactions and influence mechanisms among these community environmental factors?

RQ3: How should complex community environment transformation strategies be developed when facing real-world cases?

## 2 Literature review

In this chapter, to establish the environmental assessment attributes for the mental health of elderly migrants, the psychological stress and health impacts associated with migrant life on older adults are first examined, along with the existing research limitations concerning the mental health of this population. Subsequently, based on the social-ecological theory, the influence of the community living environment on the mental health of older adults is discussed from the perspective of individual health and the physical environment. Finally, starting from the daily behaviors that can improve the mental health of elderly immigrants, relevant community environmental elements were sorted out as the basis for the next assessment.

### 2.1 Stress in the elderly migrant population

Immigrant populations have long been a focus of academic attention (25). Early research concentrated on motivations for migration (26), preferences in migration choices (27, 28), and the two-way impact of immigration on both destination and origin countries (29). In recent years, research on older immigrants has shifted toward their lifestyles (30), identity (31), living conditions, and the degree of cultural acceptance (32, 33). The isolation of older immigrant populations stems not from long-term discrimination or prejudice based on historical, cultural, or religious factors, but rather from differentiated group attributes (34, 35). Numerous studies have shown that after leaving the workforce, older adults tend to have relatively closed social networks, making it difficult for them to establish new interpersonal relationships in the host communities. At the same time, older adults have significantly lower physical capacity and adaptive capacity to new environments compared with younger people (36), leading to their gradual social isolation and marginalization (37) and consequently to greater stress. Studies have shown that perceived stress can lead to anxiety and depressive symptoms in older immigrant populations (38). Recent research on older Chinese immigrants has primarily focused on migration characteristics (39) and health impacts (40). Due to problems such as social isolation, limited social interaction, and poor social integration, immigrant populations often experience lower levels of well-being (41). Some studies have examined the long-term impact of migration on the mental health of older adults (42) as well as the prevalence of depression among immigrants and older adults (43). In addition, studies have categorized older populations based on factors such as migration purpose and economic status and have explored the differences among these groups. For example, older adults migrating from rural areas to cities may be more vulnerable due to their relatively lower socioeconomic status and lack of social ties and social security services (44). As a long-term process, migration requires migrants to cope with various stressful events over an extended period (45).

As the economic and social status of migrant populations, particularly elderly migrants, continues to improve (30), greater attention should be paid to their health, given their high susceptibility to mental health issues. Research on this population has primarily focused on situational analysis (30–33), group characteristics (41–43), and psychological recovery (36–38). Common interventions for preventing mental illness in this group mainly involve medication (38) and social interaction (37). While such studies reveal differences among subgroups, they exhibit limitations in addressing the complex sources of stress experienced by this population. Moreover, the mental health challenges of elderly migrants cannot be resolved through singular approaches (3), necessitating more diverse perspectives to understand the factors influencing their psychological well-being.

### 2.2 Community environmental factors and mental health

Social-ecological theory offers a crucial theoretical framework for understanding the complex relationships between individual health and environmental factors. This theory emphasizes that individual health is dynamically influenced by multilevel environmental influences. Among these, the built environment, as a key physical environmental factor, is associated with residents’ health through both direct exposures (e.g., air and noise pollution) and indirect pathways (e.g., behavioral adjustments and enhanced social interactions). This framework is especially relevant for older adults because, compared with other age groups, their daily activities are more concentrated within their residential communities, rendering them more sensitive to local environmental characteristics (46).

A growing body of research has confirmed that the physical and social environments within communities are key determinants of residents’ mental health (47). Studies on the impact of the community living environment on the mental health of older adults have primarily focused on negative outcomes such as depression (48) and cognitive decline (49). Life-course epidemiology provides a theoretical framework for assessing how changes in the community environment before and after relocation affect the mental health of migrant populations (50). Current research lacks a comprehensive framework capable of encompassing the specific daily life practices of elderly migrants. The research focus remains predominantly on native elderly populations, while the distinctive behavioral needs and environmental preferences of migrant elders—such as those related to ancestral worship, intergenerational care, and adaptation to new communities—have been relatively underexplored. The present study will therefore review the environmental factors influencing the mental health of migrant populations, with a focus on everyday behaviors.

#### 2.2.1 Leisure entertainment

Existing research indicates that many individuals experience enthusiasm and enjoyment during leisure activities, which enhances subjective well-being and mitigates loneliness (51). Furthermore, the satisfaction obtained from participating in leisure activities, as well as the positive experiences gained from learning and accomplishing tasks within leisure time (52, 53), implies that educationally oriented leisure activities assist older adults in discovering meaning in life by pursuing attainable goals, thereby lowering the risk of mental illness (54). Similarly, leisure activities constitute a process of socialization, cultivating motivation to engage and a sense of social identity. This is particularly important for older adults in new environments as they develop interpersonal relationships (52). Assessment elements for this dimension include recreational facilities (55, 56), art appreciation spaces (e.g., water features and art installations), and historical or cultural sites (57, 58). In the study by Lyu J et al., many elderly respondents made new friends through recreational activities, thereby reducing their feelings of loneliness and isolation in unfamiliar environments. In addition, art appreciation spaces such as water features and art installations can lower blood pressure and cholesterol in the elderly and reduce their psychological stress through esthetic improvements (48). Furthermore, cultural and historical sites can enhance the elderly’s sense of place and local identity, and accelerate the integration of immigrant elderly into the local community (3).

#### 2.2.2 Health promotion

Appropriate outdoor physical activity can significantly mitigate age-related physical decline among older adults. Physical activity positively influences the aging process through at least three primary mechanisms: preventing common chronic diseases, enhancing cognitive function, and reducing the risk of mental illness (59, 60). Subsequent studies have further indicated that community-based physical activity also promotes social participation among older adults, improving their capacity to maintain interpersonal relationships and contribute to society (61). These findings underscore the role of community physical activity interventions in fostering age-friendly communities, facilitating social interaction, and supporting functional health in older populations (62). The assessment criteria for this dimension include the availability of health and exercise facilities (55, 63) as well as medical facilities (57, 64). Appropriate physical activity is associated with a variety of health benefits for older adults (55), such as prevention of common chronic diseases; improved sleep quality; relief of negative emotions; and prevention of depression (53). This is because physical activity increases cerebral blood flow; increases exposure to natural light; and raises neurotransmitter levels. Sufficient medical facilities are available in the community, such as counseling centers, health check-up centers, and clinics. These medical facilities provide timely safety and health support for older adults, thereby promoting positive emotions.

#### 2.2.3 Social interaction

Social interaction has been demonstrated to be an effective means of alleviating depression among older adults, enhancing their sense of value, purpose, identity, and attachment, as well as their perceived safety from crime (59). Older adults are more susceptible to social isolation in unfamiliar environments. To mitigate this risk, measures such as providing transportation options, dedicated meeting spaces, educational support, and facilitated support groups have proven effective in promoting social interaction (65, 66). Informal social contact—including frequent eye contact and greetings—helps foster stronger social bonds, and such opportunities can be shaped by various environmental factors (67, 68). The literature has identified the following evaluative elements: spaces that encourage strong social interaction (60, 69, 70) and multifunctional spaces (3, 71, 72). Spaces with strong social interaction refer to public open spaces suitable for conversation and places where dense social behavior is likely to occur. Flexible spaces accommodating activities like square dancing, outdoor picnics, and community gatherings, often taking the form of plazas, open grounds, or assembly halls (3). They offer advantages such as hosting events, functional versatility, and daily accessibility. Both different spatial forms aim to promote the well-being of older adults by providing more opportunities for contact and close social relationships to fulfill their personal responsibilities (21).

#### 2.2.4 Self-actualization

Older adults frequently encounter challenges related to their social status and roles due to age, including age-based discrimination, exclusion, and marginalization (73, 74). This phenomenon is particularly pronounced among older adults who have migrated to unfamiliar environments. Intergenerational activities can address some of the needs of older adults, children, and youth simultaneously, enhance mutual understanding and respect across generations, and help older adults regain self-esteem and a sense of self-worth (75). In the current context, technological advancements have contributed to increased social and cultural isolation among older adults, exacerbating feelings of alienation (76). This dimension comprises two items: intergenerational activities (e.g., children’s playgrounds and parent–child activity centers) (3) and skill development (e.g., senior universities, community classes, and re-employment centers) (55). Qiu Z et al.’s research found that older adults who care for their grandchildren engage in a significant amount of activity, primarily within the community. Therefore, establishing children’s recreational facilities within the community is essential to meeting the daily intergenerational activity needs of immigrant older adults (3). Furthermore, lifelong learning and education are crucial for the social and cultural integration of older adults (77), and many communities have established universities for seniors and vocational training centers to help older adults integrate into new environments (78).

#### 2.2.5 Daily tasks

Due to temporal and spatial constraints, household chores and daily routines represent the two most prevalent types of activities among older immigrants (3), a pattern that closely parallels that of local older adults (79). One study revealed that in rural and densely populated urban communities, older adults often walk to visit friends or shop, whereas in suburban areas with poor accessibility, few destinations are within walking distance, leaving older adults largely confined to their homes (80). Furthermore, research indicates that interaction with pets enhances the quality of life of older adults and effectively mitigates depression, anxiety, cognitive impairment, and behavioral and psychological symptoms of dementia (BPSD). Regarding physical health, sustained interaction with pets has been associated with significant increases in physical activity and improvements in blood pressure and heart rate variability, demonstrating sustained physical health benefits (81). Therefore, the factors considered in this dimension are ancestral worship sites (including religious belief sites and places of worship), transportation, pet facilities, and convenient living facilities (e.g., grocery stores, farmers’ markets, pharmacies, and package pickup points). In East Asian communities, there are often places of folk belief. Elderly immigrants have strong feelings for their hometowns and often need to satisfy their daily spiritual needs by worshipping and burning incense on special festivals. Participating in religious ceremonies helps alleviate homesickness (79). The diversity of transportation methods determines the scope of daily travel for elderly immigrants (3). Convenient transportation helps the elderly quickly familiarize themselves with the urban environment and expand their social circle, adding more possibilities to their daily activities. Adequate amenities are crucial for daily activities; the convenience of everyday facilities allows older adults to combine walking with other daily social activities, leading to more frequent visual contact and greetings (82).

#### 2.2.6 Environmental exposure

This dimension mainly includes landscape vegetation, acoustic environment quality, and ambient temperature. Contact with nature can promote health and well-being (83, 84). For older adults, it can bring positive effects such as improved physical and mental health, increased neurotransmitter levels, and stress reduction. Possible reasons for this positive impact include improved air quality, stimulation of physical activity, increased social interaction, and stress recovery and relief (85). In addition, environmental noise exposure can, through fatigue, lead to anxiety among community residents (86). For older immigrants who have just arrived in a new environment, it can also cause sleep disorders and mental disorders such as bipolar disorder (87). Furthermore, research indicates that vulnerable populations, such as older adults, are more sensitive to variations in environmental temperature (88). In comparison with younger individuals, older adults typically exhibit reduced cutaneous thermal sensitivity and greater difficulty in maintaining core body temperature during cold exposure (89). Low temperatures adversely affect the central nervous system in older adults and may accelerate the onset or progression of mental disorders (90).

## 3 Research design and methods

**Figure 1:**
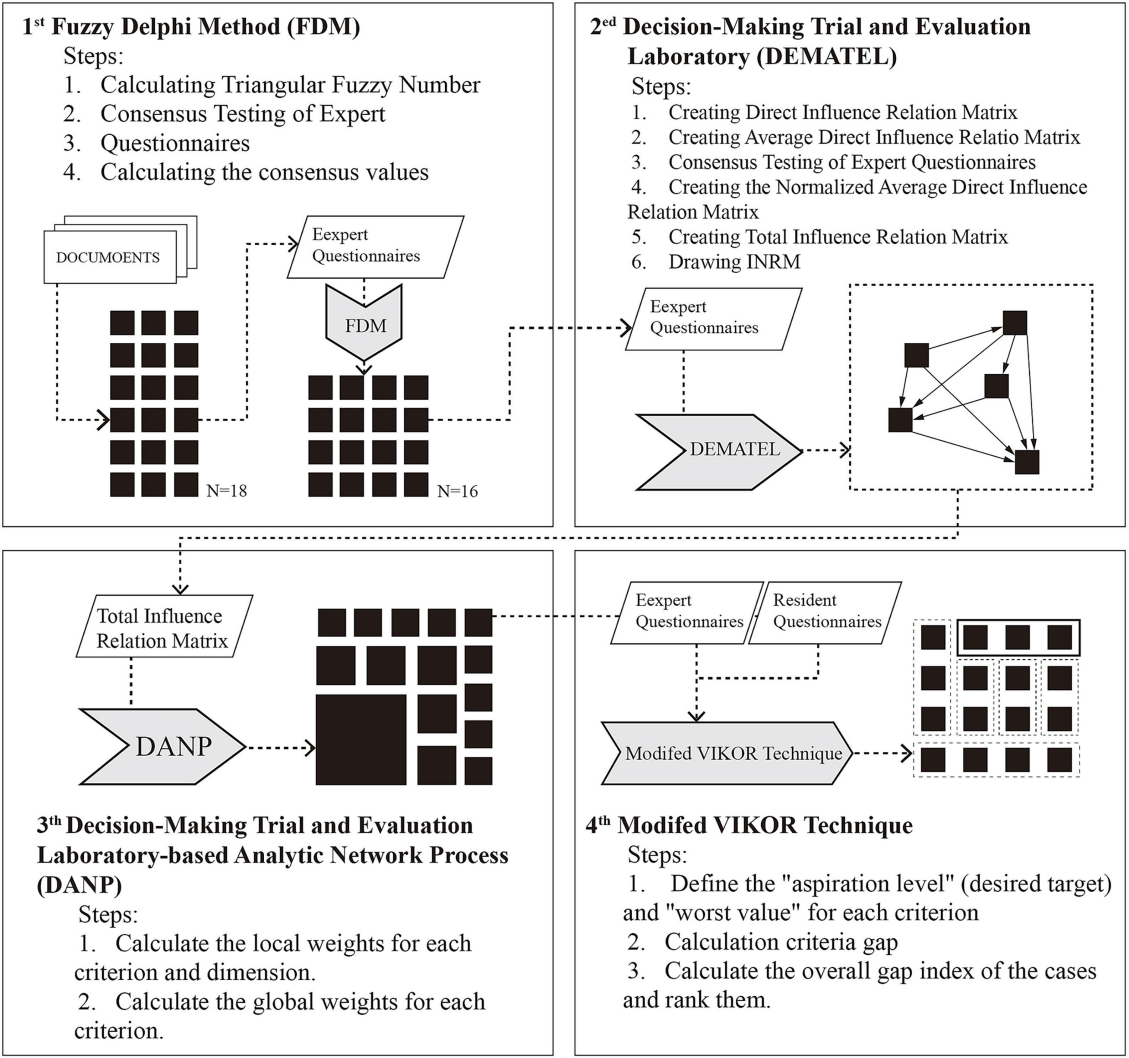
Flowchart of multi-attribute decision model.

The methodological framework of this study comprises three stages (Figure 1). In the first stage, a community environmental factor scale was developed by initially identifying factors from the literature on the daily behaviors of the older floating population, which were then screened and optimized using the Fuzzy Delphi Method (FDM) to incorporate expert judgment, resulting in a systematic indicator system. In the first stage, a community environmental factor scale was developed by adopting the daily behavioral needs of the older floating population as the classification framework. Specific indicators were identified from the literature and categorized into these dimensions, and then screened and optimized using the Fuzzy Delphi Method (FDM) to incorporate expert judgment, resulting in a systematic indicator system. In the second stage, the DANP-V model (91, 92) was adopted: first, the DEMATEL method was used to administer questionnaires to domain experts and, based on the results, to compute the relation matrix of the total influence of dimensions (TD) and the total influence of criteria (TC); then the DANP method was used to derive the criteria influence weights from the total-influence relation matrix. Finally, a modified VIKOR method was used to identify problematic criteria in this study; the larger the gap value of a criterion, the more severe the defect (93, 94). Following this methodological pathway, strategies for improving community environments beneficial to elderly migrants were established.

### 3.1 Fuzzy Delphi method

The Delphi technique, initially formulated by Dalkey (95), represents a systematic methodology aimed at synthesizing expert judgments to facilitate consensus formation. Subsequently, Ishikawa et al. enhanced this approach through the integration of cumulative frequency distributions and fuzzy set integrals (96), leading to the establishment of the Fuzzy Delphi Method (FDM). Further refinement was contributed by Jeng (97), who introduced a gray zone identification technique utilizing double-triangular fuzzy numbers to optimize the evaluation of consensus convergence among experts (97). It enables experts to provide more nuanced responses, expressing varying degrees of agreement or disagreement with a particular statement or question (96).

### 3.2 DANP-V model

The DANP-V model is a hybrid technique in multi-attribute/multi-criteria decision making (MCDM). It combines three techniques: the DEMATEL method, the DANP method, and the VIKOR method (21). Chosen over other MCDM approaches for its ability to handle the systemic nature of community environments where factors form an interconnected whole, this method aims to construct and solve decision and planning problems involving multiple conflicting criteria (91). It possesses the following characteristics: first, unlike standard methods that assume linear independence, it captures the complex, multi-layered interrelationships among criteria (98); second, it enables program selection in situations with conflicting attributes (99) and uses the aspiration level as a benchmark to avoid “picking the best apple from a barrel of rotten apples” replacing the limitations of past evaluation methods based solely on relative standards (21); finally, distinct from methods that only strictly rank options, this approach identifies specific gaps to formulation feasible and systematic improvement strategies (100). In previous studies, this method has been used to assess and improve public open spaces for older adults and has successfully developed stepwise and effective environmental improvement strategies (21).

## 4 Case studies and data analysis

This section details the selection, data collection, and analysis processes for empirical cases. Furthermore, based on INRM and performance evaluation results, it focuses on discussing strategies for improving the community environment.

### 4.1 Resource identification initiative

Based on a literature review, the researchers employed the Fuzzy Delphi Method (FDM) to evaluate the significance of community environmental factors associated with the mental health of older immigrants, aiming to identify core environmental factors affecting this population’s mental well-being. The study was conducted in China, and the questionnaire was translated into Chinese prior to the formal survey to facilitate expert opinion collection. From September 21 to October 1, 2025, a total of 27 FDM questionnaires were distributed online and offline to experts in landscape design and urban spatial studies. All questionnaire items were rated using a 10-point Likert scale, ranging from 1 (completely unimportant) to 10 (extremely important). Experts were asked to provide both a “conservative” and an “optimistic” rating for each attribute. After reviewing background information and survey instructions, participating experts first completed a personal information section. All experts were also invited to submit written comments and suggestions for each item. In the first round of the survey, 4 questionnaires were deemed invalid due to incomplete responses or inconsistent values, while the remaining 23 questionnaires were considered valid. To determine whether a criterion should be retained, it is necessary to set a benchmark for consensus values. In this study, the benchmark was established at 4 out of 10, meaning that criteria with scores at or above this threshold were kept, while those below were excluded. The final FDM results identified 16 evaluation elements across 6 dimensions for subsequent analysis (Table 1).

**Table 1:** Environmental factors test results.

Dimension	Criteria	Descriptions	References
Leisure entertainment (*D_1_*)	Entertainment Venues (*C_11_*)	Consumer entertainment venues with social and cultural attributes. Elderly migrants in less developed regions desire to experience advanced facilities or lifestyle activities typical of developed areas/younger populations, using them as a medium for social interaction. Examples: Karaoke bars, shopping malls, retail complexes, cinemas, cafes, dance halls, chess/card rooms.	(51, 55, 56)
	Art Viewing Space (*C_12_*)	Public art and scenic spots provide temporary respite and adjustment periods for elderly migrants, essentially offering visual solace to alleviate pressure. Examples: Pleasing natural landscapes, cultural landmarks, public art installations.	(21, 55)
	Historical & Cultural Sites (*C_13_*)	Addresses needs through both tourism and social interaction. Examples: Local temples/places of worship, commemorative plaques, historical relics.	(57, 58)
Health promotion (*D_2_*)	Recreation & Fitness Facilities (*C_21_*)	Regular physical activity can help seniors regain vitality and improve sleep quality. Examples: Sports fields, table tennis tables, gateball courts, recreational facilities, fitness equipment.	(55, 63)
	Healthcare Facilities (*C_22_*)	Medical facilities meet the elderly’s need for a sense of safety and security. Examples: Emergency stations, first-aid kits, clinics, community health screening centers.	(57, 64)
Social interaction (*D_3_*)	Venues for Strong Social Interaction (*C_31_*)	Examples: Public open spaces suitable for conversation, small-scale intimate conversation areas, flexible spaces for hosting events and expanding social networks.	(60, 69, 70)
	Multi-Functional Spaces (*C_32_*)	Flexible spaces accommodating activities like square dancing, outdoor picnics, and community gatherings, often taking the form of plazas, open grounds, or assembly halls. They offer advantages like hosting events, functional versatility, and daily accessibility.	(3, 71, 72)
Self-actualization (*D_4_*)	Intergenerational Activities (*C_41_*)	A significant proportion of elderly migrants have intergenerational caregiving responsibilities. Environments with child-friendly recreational attributes are suitable for activities with grandchildren, fulfilling childcare needs. Examples: Children’s playgrounds, parent–child activity centers.	(3, 75)
	Skill Development (*C_42_*)	Educational institutions, particularly non-degree programs, provide learning opportunities for the elderly, allowing them to regain a sense of purpose and value. Examples: University for the aged/elderly college, community classes, senior re-employment centers.	(55, 76, 78)
Daily tasks (*D_5_*)	Ancestral Worship Sites (*C_51_*)	Elderly migrants often have strong attachment to their hometowns. Participation in rituals like Qingming Festival or Ghost Festival helps alleviate homesickness. Examples: Religious sites, spaces for burning ritual offerings.	(3, 79)
	Living Convenience Facilities (*C_52_*)	Within the community, elderly migrants rely on daily facilities to maintain household operations. Examples: Pharmacies, package pickup points, grocery stores, fresh food markets, government service centers.	(25, 57)
	Transportation & Mobility (*C_53_*)	Diverse transportation options help elderly migrants explore, familiarize themselves with the city and their neighborhood, and expand their daily activity range.	(3)
	Pet-Friendly Facilities (*C_54_*)	Many elderly migrants lack family companionship in their new environment, shifting emotional attachment to pets, necessitating supporting facilities. Examples: Pet waste stations, pet parks.	(82)
Environmental exposure(*D_6_*)	Landscape vegetation (*C_61_*)	Plants and trees with suitable location, collocation, and seasonal interest	(83, 84)
	Acoustic Environment Quality (*C_62_*)	Noise is a contributor to depression, anxiety, stress, fatigue, headaches, and sleep disturbances in the elderly, primarily from traffic, residential renovation, and commercial noise.	(87)
	Ambient Temperature (*C_63_*)	Cold environments are associated with an increased probability of depression in the elderly. Examples: Considerations for wind protection, shading, sunlight control.	(89, 90)

### 4.2 Study area

**Figure 2:**
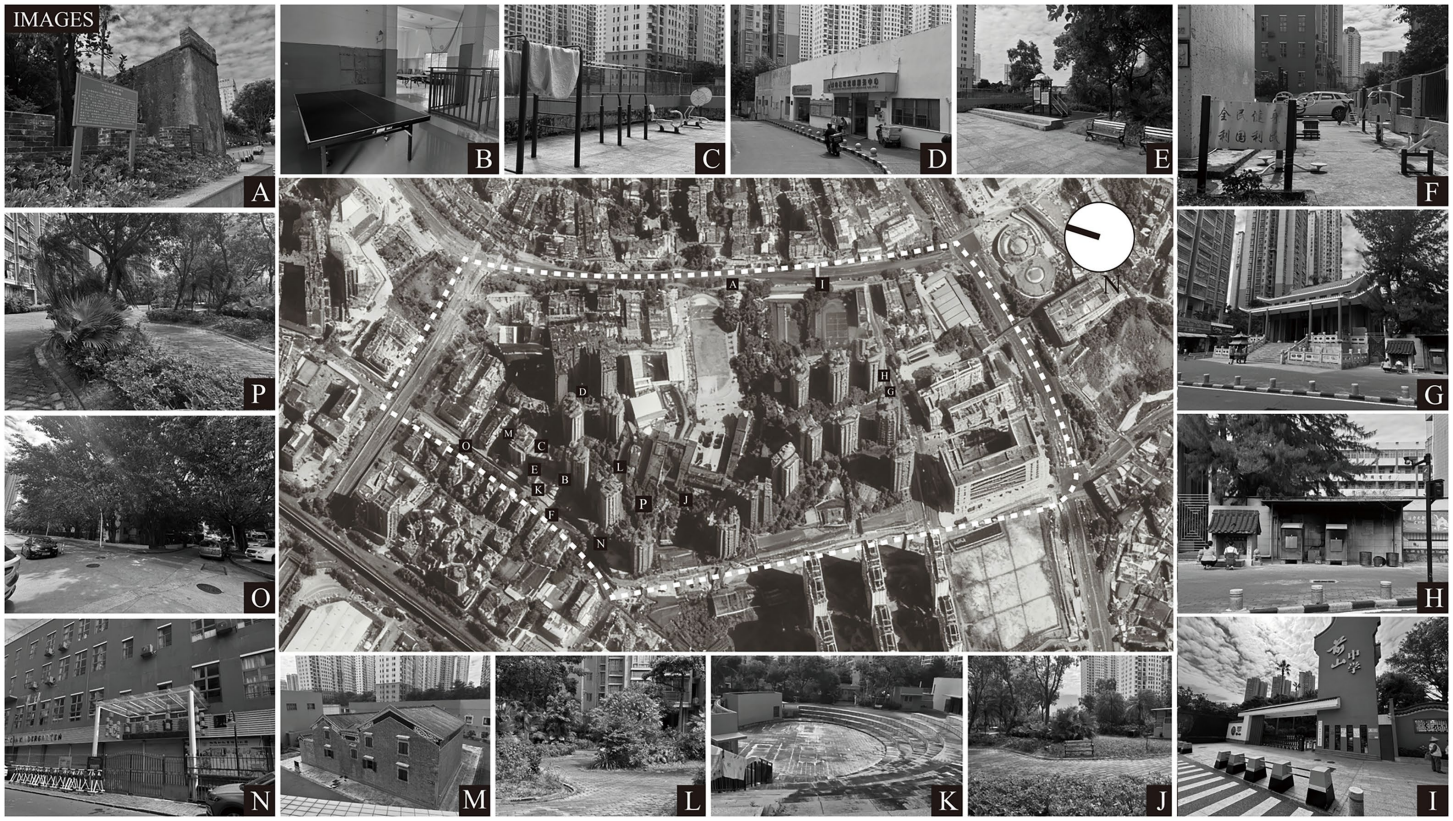
Community environment images.

Zhuhai has entered a stage of mild population aging, with registered residents aged 60 and above accounting for 14.94% of the population by the end of 2023. Known as a “retirement paradise,” the city attracts a considerable number of retired older adults from across China (101). In 2019 alone, over 100,000 people from other provinces in China migrated to Zhuhai (102). Therefore, the city has placed growing emphasis on its elderly immigrant population (102). This study selected Qianshan Community in Zhuhai, a community with a high proportion of immigrants and a serious aging problem. First, the community is a typical settlement for migrant workers and their accompanying family members (including the elderly), offering a low cost of living and well-developed surrounding facilities, including two schools (Figure 2I), one kindergarten (Figure 2N), supermarkets, green spaces, and a swimming pool. The floating population accounts for 60% of the community, exceeding the permanent resident population. Second, to address aging-related issues, Qianshan Community has made numerous environmental improvements that take into account the behavioral needs of the elderly, including daytime care (Figure 2D), psychological counseling (Figure 2B), cultural and recreational activities (Figure 2F), and religious beliefs (Figures 2G,H). Furthermore, Zhuhai is actively building “cross-border elderly care” and “Greater Bay Area elderly care” models to improve the community environment for elderly immigrants and is promoting the construction of a “15-min elderly care service circle” to attract more elderly migrants from other regions (103).

Therefore, given the rapid growth of the aging population, the large proportion of immigrants, and the diverse environmental characteristics of Qianshan Community, this site was chosen as a case study to investigate environmental improvement strategies to promote the mental health of older immigrants.

### 4.3 Data collection

This section delineates the data collection procedure and the selection of participants, comprising an expert panel and local residents. The study employed a DEMATEL questionnaire administered to experts to investigate the interrelationships among 16 criteria. A separate VIKOR questionnaire was distributed to both the expert and resident groups. Given that relying exclusively on either expert judgments or user feedback in decision-making is frequently debated, this study additionally compared the outcomes from the two groups to analyze potential disparities in satisfaction levels.

The respondents in the DEMATEL expert survey were drawn from a range of sectors, including various industries, government agencies, and academic institutions, and possessed an in-depth understanding of both the theoretical and practical dimensions of the research topic. Their expertise covered fields such as older adult mental health, migration management, community environment development, landscape planning, and the eldercare industry. Prior to completing the questionnaire, the researchers explained its purpose and procedures to the experts and addressed any questions they raised during the process. A total of 11 questionnaires were distributed, of which 9 were valid and 2 were invalid due to incomplete responses. Consistency in expert judgment is a prerequisite for the validity of expert data. Three days after experts completed the DEMATEL questionnaire, researchers conducted a reliability test on the results by distributing the questionnaire repeatedly. By comparing the relationship matrices obtained from the two surveys, the absolute difference was 4.791. This value is far less than the theoretically maximum possible difference, indicating that the cumulative effect of the differences among all 256 paired data points is extremely low. The average difference of a single relationship value is less than 0.019, confirming the consistency of expert judgment.

For the VIKOR questionnaire, a set of 14 questionnaires was distributed via email to an external panel of experts, from which 10 valid responses were obtained. All participating experts were based in Zhuhai and possessed comprehensive knowledge of the study area. As landscape planners with some background in aging and geriatrics, they were well-qualified to evaluate the relevant environmental factors. Among them, eight experts had visited Qianshan Community in person; the remaining two, who had not visited the site, were briefed with videos and photographs taken by the research team to ensure their familiarity with the location. Consequently, all experts were deemed competent to complete the questionnaire, which covered 16 environmental factors, each to be rated on a satisfaction scale from 1 to 10, where 1 indicated extreme dissatisfaction and 10 indicated extreme satisfaction.

From September 15 to 26, 2025, researchers conducted face-to-face VIKOR questionnaire surveys with older migrants in Qianshan Community. Before conducting the questionnaire survey, researchers asked respondents if they were immigrants to confirm their eligibility for participation. They also obtained their consent before they completed the questionnaire. Respondents rated their satisfaction with 16 community environmental evaluation factors based on their own experiences. Researchers distributed the questionnaire randomly during two time periods (08:00–11:00 and 03:00–18:00). The community environment is relatively closed, and most respondents resided in Qianshan Community. Before completing the questionnaire, researchers confirmed whether they met the study criteria and obtained their consent. Participation was entirely voluntary, and older adults could refuse to take part in the study. During distribution, researchers prioritized older adults who were resting, reading, engaging in light exercise, or participating in intergenerational activities. One question (“How long have you been settled in Zhuhai?”) was removed from the questionnaire, as many respondents had forgotten or were unwilling to answer it. In addition, for respondents with visual impairments, researchers read the questionnaire aloud and assisted them in completing it. A total of 164 questionnaires were collected, of which 24 were invalid due to a large number of unanswered items, resulting in 140 valid questionnaires (Table 2). Among the respondents, 66 (47.1%) were male and 74 (52.9%) were female. The majority (58.6%) of respondents held non-local household registration. Given the case study community, the questionnaire results exhibited diversity and high saturation, encompassing elderly immigrants of different ages and genders. Respondents were all long-term residents of the community, familiar with its environment, and their opinions on the modification of environmental elements in the case study were considered reliable. We assessed the adequacy of the sample size using the formula provided by Cochran (104). The analysis relied on the 140 valid responses collected. For this calculation, we assumed maximum variability and applied a 95% confidence level. This yielded a margin of error of 8.28%. Large-scale studies typically require a 5% margin. However, established methodological guidelines suggest that a margin of up to 10% is acceptable in specific contexts (105, 106). Such a threshold is particularly relevant for descriptive research involving hard-to-reach demographic groups. Therefore, the sample size of 140 valid questionnaires collected from the elderly immigrant population in this study was deemed reasonable.

**Table 2:** Respondent information segmented by demographic characteristics (*N* = 140).

Variables		N	Percentage (%)
Gender	Male	66	47.1
	Female	74	52.9
Age	60–65	25	17.9
	66–70	71	50.7
	71–75	23	16.4
	76–80	13	9.3
	81 and above	8	5.7
Living conditions	Living alone	19	13.6
	Living with spouse	45	32.1
	Living with children	68	48.6
	Nursing home	8	5.7
Household registration(Zhu Hai)	Yes(Zhu Hai)	58	41.4
	No	82	58.6
Monthly income status	≤3000元	31	22.1
	3,000-6,000元	69	49.3
	≥6,000元	40	28.6
Is your spouse still alive?	Yes	95	67.9
	No	45	32.1

## 5 Results and discussion

### 5.1 Data result analysis

**Figure 3:**
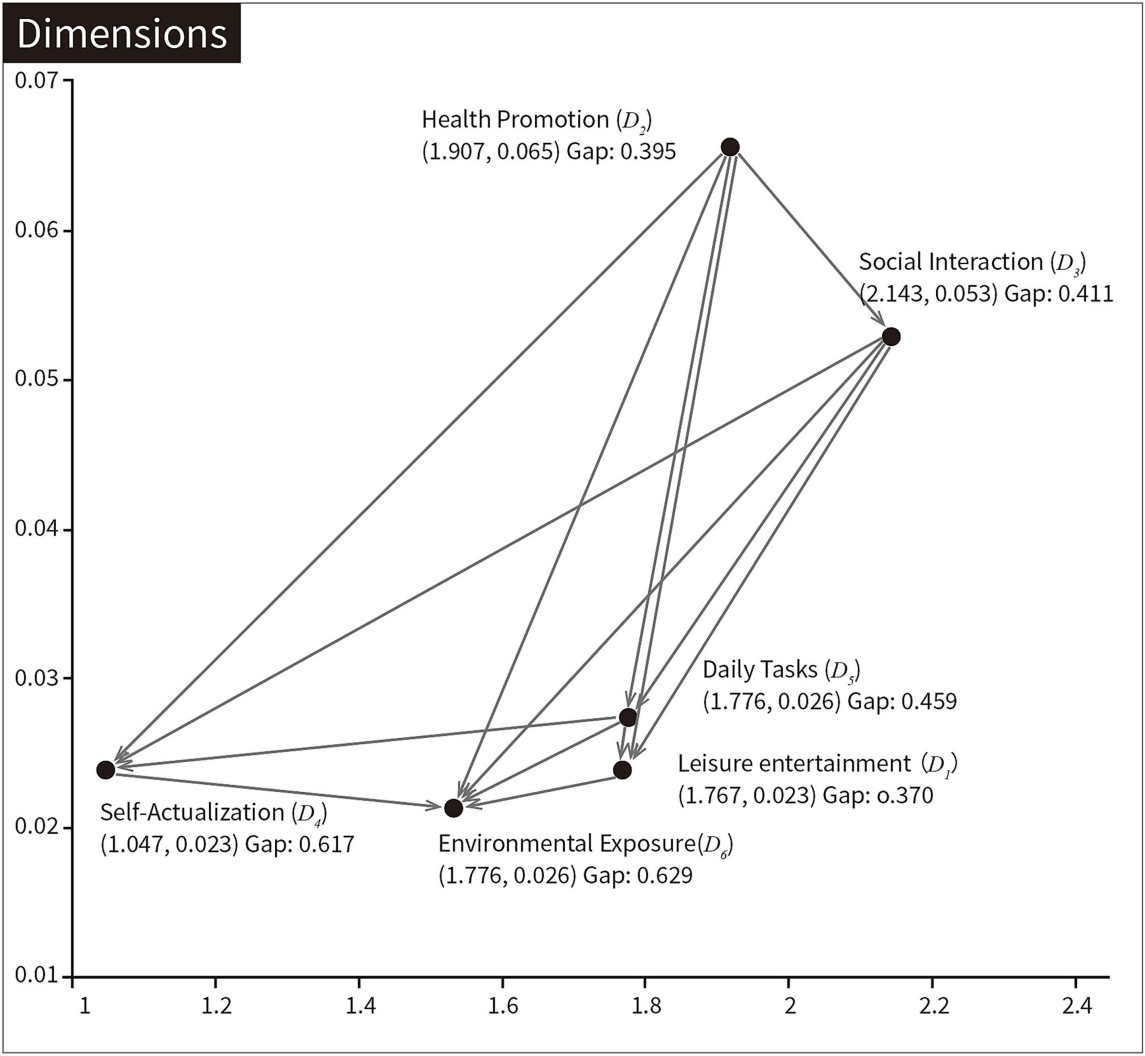
The INRM (influential network relation map) of total influence relationships.

Figure 3 illustrates the relationships of influence among the dimensions. Self-Actualization (*D_4_*) is influenced by Health Promotion (*D_2_*), Social Interaction (*D_3_*), and Daily Tasks (*D_5_*). Environmental Exposure (*D_6_*) is influenced by the other five dimensions. The influence priority order of the six dimensions is *D_2_*, *D_3_*, *D_5_*, *D_1_*, *D_4_*, and *D_6_*. Entertainment Venues (*C_11_*) denotes the most important factor influencing Leisure entertainment (*D_1_*); Recreation & Fitness Facilities (*C_21_*) is the most important factor influencing *D_2_*; Venues for Strong Social Interaction (*C_31_*) is the most important factor influencing *D_3_*; Intergenerational Activities (*C_41_*) denotes the most important factor influencing *D_4_*; Transportation & Mobility (*C_53_*) is the most important factor influencing *D_5_*; and Ambient Temperature (*C_63_*) denotes the most important factor influencing *D_6_*.

Table 3 presents a systematic assessment of the impact of the Qianshan Community environment on the mental health of older immigrants. Performance scores and gap values for each dimension and criterion are calculated based on the DANP-V model. The table includes six dimensions [Leisure entertainment (*D_1_*), Health Promotion (*D_2_*), Social Interaction (*D_3_*), Self-Actualization (*D_4_*), Daily Tasks (*D_5_*), and Environmental Exposure (*D_6_*)] and 16 criteria. It reports two gap values (deviations from ideal values, where larger gap values indicate more severe problems) derived separately from the expert group and the older adult group, along with a composite value that integrates data from both groups.

**Table 3:** The performance evaluation of the case study using VIKOR.

Dimensions/criteria	Influential weights (IWs)		Elderly		Experts		Integration	
	Local weight	Global weight	Performance	Global weight	Performance	Global weight	Performance	Global weight
Leisure entertainment (*D_1_*)	0.150		6.240	0.376	7.207	0.279	6.304	0.370
Entertainment Venues (*C_11_*)	0.436	0.065	7.157	0.284	8.000	0.200	7.213	0.279
Art Viewing Space (*C_12_*)	0.364	0.055	5.186	0.481	6.300	0.370	5.260	0.474
Historical & Cultural Sites (*C_13_*)	0.200	0.030	6.157	0.384	7.300	0.270	6.233	0.377
Health Promotion (*D_2_*)	0.125		5.924	0.408	7.854	0.215	6.053	0.395
Recreation & Fitness Facilities (*C_21_*)	0.530	0.066	4.179	0.582	7.200	0.280	4.380	0.562
Healthcare Facilities (*C_22_*)	0.470	0.059	8.036	0.196	8.700	0.130	8.080	0.192
Social Interaction (*D3*)	0.150		5.767	0.423	7.571	0.243	5.887	0.411
Venues for Strong Social Interaction (*C_31_*)	0.608	0.091	6.171	0.383	8.000	0.200	6.293	0.371
Multi-Functional Spaces (*C_32_*)	0.392	0.059	5.171	0.483	7.000	0.300	5.293	0.471
Self-Actualization (*D4*)	0.125		3.693	0.631	5.717	0.428	3.828	0.617
Intergenerational Activities (*C_41_*)	0.510	0.064	3.229	0.677	6.200	0.380	3.427	0.657
Skill Development (*C_42_*)	0.490	0.061	4.164	0.584	5.200	0.480	4.233	0.577
Daily Tasks (*D_5_*)	0.225		5.346	0.465	6.364	0.364	5.414	0.459
Ancestral Worship Sites (*C_51_*)	0.192	0.043	2.793	0.721	3.900	0.610	2.867	0.713
Living Convenience Facilities (*C_52_*)	0.332	0.075	7.164	0.284	7.900	0.210	7.213	0.279
Transportation & Mobility (*C_53_*)	0.339	0.065	6.186	0.381	6.900	0.310	6.233	0.377
Pet-Friendly Facilities (*C_54_*)	0.137	0.026	2.157	0.784	2.900	0.710	2.207	0.779
Environmental Exposure (*D_6_*)	0.225		3.553	0.645	5.863	0.414	3.707	0.629
Landscape vegetation (*C_61_*)	0.345	0.078	5.179	0.482	6.900	0.310	5.293	0.471
Acoustic Environment Quality (*C_62_*)	0.216	0.049	3.236	0.676	6.000	0.400	3.420	0.658
Ambient Temperature (*C_63_*)	0.439	0.099	2.579	0.742	5.000	0.500	2.740	0.726
Total Performance			4.942		6.534		5.048	
Total Gap (ratio)				0.506		0.347		0.495

Overall, Table 3 shows a total gap value of 0.495, indicating that the community environment as a whole has not reached an ideal level. Among the six dimensions, Environmental Exposure (*D_6_*) has the largest gap value (0.629), mainly involving Landscape vegetation (*C_61_*; 0.471), Acoustic Environment Quality (*C_62_*; 0.658), and Ambient Temperature (*C_63_*; 0.726). The main reason for this phenomenon is likely the high dependence of residents’ daily lives on the community environment. Prolonged negative acoustics, significant climate differences from their hometowns, and physical vulnerability all contribute to exacerbating the mental health problems of elderly migrants. Self-Actualization (*D_4_*) has the second-largest gap value (0.617). The following indicators have relatively large gap values: Ambient Temperature (*C_63_*; 0.726), Ancestral Worship Sites (*C_51_*; 0.713), and Acoustic Environment Quality (*C_62_*; 0.658). The following indicators have relatively small gap values: Healthcare Facilities (*C_22_*; 0.192), Living Convenience Facilities (*C_52_*; 0.279), and Entertainment Venues (*C_11_*; 0.279).

### 5.2 Case improvement strategy

To address the issues identified in this case, improvement strategies will be developed and a comprehensive analysis will be conducted based on the assessment results from both the expert panel and the older residents panel. The gap values across the six dimensions indicate significant room for improvement in environmental factors affecting the mental health of older immigrants in this case. The gap value for Leisure and Entertainment (*D_1_*) is relatively small, suggesting that this dimension is regarded as more essential and receives comparatively greater support from decision-makers. As evident from the community environment (as shown in Figure 2), the area possesses a richer historical and cultural heritage compared to other newly built communities, featuring historical sites (Figure 2A) and cultural buildings (Figure 2M). The area also offers diverse recreational facilities for residents (Figures 2B,C,F), which may account for the smaller gap value in *D_1_*. Priority should be given to improving areas with large gap values: Pet-Friendly Facilities, Ambient Temperature, Ancestral Worship Sites, and Acoustic Environment Quality. Previous community environment improvement programs have addressed these gaps sequentially, from largest to smallest. In contrast, the DANP-V method tends to adopt a systemic approach to tackle root causes (21). For example, in this community, Pet-Friendly Facilities (*C_54_*; gap value 0.779), which exhibits the largest gap, should be the primary focus for improvement. Pets serve as a common means for older adults to relieve psychological stress (107), and this case study community also demonstrates a high rate of pet ownership. Walking dogs and managing pet waste are major concerns for these older adults, as they often restrict dog walking to within the community due to health and time constraints. Additionally, Ancestral Worship Sites (*C_51_*) also show a substantial gap. Older adults in China maintain the tradition of “ancestor worship,” a practice that persists even after migration (108). The largest gap value among all factors is for Pet-Friendly Facilities (*C_54_*; 0.779). This significantly high gap value underscores a critical behavioral characteristic of elderly migrants: the reliance on pets for emotional companionship to cope with social isolation in a new environment. The lack of dedicated facilities, such as pet waste stations, directly conflicts with this adaptive behavior, creating spatial conflicts and practical difficulties in their daily routines. In the places to which they have migrated, this behavior is considered part of older adults’ psychological adaptation strategies (109). This notable gap reflects the importance of cultural rituals as a psychological adaptation strategy for elderly migrants. The act of ancestor worship serves as a crucial link to their place of origin, helping to alleviate homesickness. Its inaccessibility in the new community thus represents a significant barrier to their cultural and psychological well-being. According to the DANP-V model, satisfaction with Transportation & Mobility (*C_53_*) and Living Convenience Facilities (*C_52_*) influences *C_54_* and *C_51_*. In such cases, policymakers should prioritize investing resources in addressing the root causes of these issues. The high gap value for Pet-Friendly Facilities reflects an urgent need for emotional companionship among older immigrants; however, its effectiveness is highly dependent on community accessibility and functional support (110). If there are obstacles in *C_53_*—such as discontinuous walking paths or insufficient rest areas—the range of activities available to older people with pets becomes limited, which may exacerbate spatial conflicts, including issues related to pet waste disposal. Similarly, the availability of Living Convenience Facilities directly affects the feasibility of establishing Ancestral Worship Sites; if the community lacks convenient grocery stores or religious supply stores, older adults may face difficulties in acquiring the necessary supplies for worship.

The results revealed in the model also provide empirical support for the social-ecological theory. The mental health of older immigrants is built upon a range of environmental factors and can be improved through community-based environmental factors. Improving environmental factors with significant problems, such as Pet-Friendly Facilities (*C_54_*) and Ancestral Worship Sites (*C_51_*), will effectively alleviate individual mental health stress by promoting behavioral change. This also confirms the interrelationships among environmental elements. For instance, the large gap in Pet-Friendly Facilities (*C_54_*) is not merely an isolated issue but is influenced by the accessibility of Transportation & Mobility (*C_53_*). From an Environmental Design Theory perspective, if walking paths (*C_53_*) are discontinuous, the utility of pet facilities (*C_54_*) is diminished, creating a conflict between the elderly migrants’ need for emotional companionship and the environmental constraints. Similarly, the provision of Ancestral Worship Sites (*C_51_*) is constrained by the availability of Living Convenience Facilities (*C_52_*). This interplay shows that a failure in one part of the social-ecological system can disrupt a culturally significant coping mechanism, thereby exacerbating mental health risks. Therefore, improvements must target these systemic linkages rather than isolated symptoms.

### 5.3 Performance evaluation and gap analysis

This study conducted a comprehensive analysis of survey data collected from experts and elderly participants to derive performance scores and gap values. The results indicated that the gap values obtained from the questionnaire for elderly migrants were generally larger than those from the questionnaire for design experts. The researchers suggest that these discrepancies may be attributed to the following reasons: First, elderly migrants had resided in the area for a longer duration and possessed more direct experience with the case, leading to greater environmental exposure. Second, as most respondents originated from other regions, their living habits have long been shaped by their places of origin, and they continue to face challenges in adapting to the local community’s lifestyle. Previous studies have confirmed that strong place attachment significantly reduces environmental adaptability among elderly migrants (111, 112). Finally, the distinct needs of the elderly migrant population may also contribute to this outcome. This questionnaire evaluated environmental factors influencing the mental health of elderly migrants, whose behavioral needs differ from those of local elderly individuals; for example, they place greater emphasis on ancestral worship and on keeping pets to alleviate psychological stress.

Furthermore, when comparing the two sets of results, the study found that the ranking patterns of the various criteria between the designer experts and the elderly immigrants were largely consistent, particularly for the top three ranked criteria. However, a significant discrepancy emerged between the two groups’ ratings for Intergenerational Activities (*C_41_*). During interviews, researchers noted that this divergence may be related to the relatively limited variety of intergenerational activity venues and the aging or outdated condition of community facilities, which, after long-term use, diminished users’ sense of novelty and engagement. Expert assessments, on the other hand, tended to focus more on the presence of intergenerational activity spaces and the completeness of their facilities. Given the distinct circumstances of elderly immigrants compared with other groups, greater emphasis should be placed on public participation when enhancing the community environment, and efforts should be made to encourage immigrants to contribute opinions and suggestions for improving age-friendly community environments. By integrating the evaluation results of experts and users, decision-makers can more accurately identify the needs and priorities of user groups.

## 6 Conclusion

This study systematically evaluated, using the DANP-V model, the mechanisms through which the community environment affects the mental health of older immigrants, revealing the intrinsic connections among environmental factors and their differentiated effects on mental health, with the aim of improving the quality of life and social adaptability of older immigrants.

### 6.1 Theoretical contributions

Older immigrants face challenges related to aging and mobility limitations, which may lead to social isolation and negatively affect their mental health. This study supports the view that the community environment is closely related to immigrant mental health (13). The findings provide additional effective avenues for promoting the mental health of older immigrants from the perspective of environmental factors. This study deepens and expands the interdisciplinary field of environmental gerontology and migrant health research by focusing specifically on the elderly migrant population. Unlike most existing studies that concentrate on local elderly populations or are situated in Western social contexts, this study identifies and validates unique environmental elements, such as Pet Friendly Facilities (*C_54_*) and Ancestral Worship Sites (*C_51_*) that are crucial for the mental health of elderly migrants. The study starts from the behavioral characteristics that influence the mental health of older immigrants and focuses on the environmental factors that give rise to these behaviors, thereby responding, to some extent, to previous discussions on how the behavior patterns of older immigrants are constrained by environmental factors (3). These findings reveal significant population heterogeneity in the relationship between environment and health, indicating that community environmental designs suitable for the general elderly population may not meet the specific needs for cultural adaptation and emotional attachment among elderly migrants. Consequently, by introducing the daily behavior perspective of elderly migrants, this study provides a more refined theoretical explanation for understanding the mechanisms through which the built environment impacts mental health.

### 6.2 Methodological contributions

At the methodological level, this study demonstrates the applicability and effectiveness of the DANP-V model, a hybrid Multiple Criteria Decision Making (MCDM) model, in community environmental assessment and improvement strategy research. Compared to traditional statistical analysis models that often assume the independence of evaluation criteria, this method effectively captures and quantifies the complex interdependencies among various elements in the community environment. By constructing an Influential Network Relation Map, this study not only determines the weights of environmental factors but also reveals their inherent causal relationships. This provides a methodological tool for developing systematic improvement strategies that address root causes, overcoming the limitation of previous studies that could only offer isolated recommendations.

### 6.3 Practical contribution

At the practical level, this study provides a clear priority sequence and concrete action pathways for community governance and spatial optimization targeting elderly migrants. The results indicate that policymakers and environmental designers should move beyond a singular focus on conventional facilities like recreation and fitness. Instead, priority should be given to addressing dimensions that have a profound impact on the mental health of elderly migrants but have long been overlooked, such as Environmental Exposure (*D_6_*) and software/hardware support for Self-Actualization (*D_4_*). The assessment of the case community shows that improving the accessibility of Pet-Friendly Facilities (*C_54_*) and the convenience of Daily Tasks (*D_5_*) has a leverage effect on alleviating loneliness and promoting social integration among elderly migrants.

### 6.4 Research limitations

The study has limitations that can provide guidance and experience for future research. First, data collection was limited to older immigrants in the Qianshan community of Zhuhai and focused on internal migrant groups, which may restrict the generalizability of the findings. While the research process and assessment framework can serve as a reference for studying environmental improvement strategies in many immigrant communities, they may not be applicable to all settings. Second, users from different sociocultural backgrounds within the immigrant population may have varying needs and perceptual preferences, and how to further segment participants is another challenge for future research. The process from immigration to integration is long and multidimensional. This study aimed to explore the most pressing psychological issues of older immigrants from an environment–behavior perspective. Undeniably, the social integration of older immigrants cannot be resolved through a single approach. Future research should consider incorporating more relevant factors (e.g., cultural identity) into environment–behavior strategies. Finally, in addition to spatial strategies, temporal changes should also be taken into account. Future research could adopt multi-stage assessments to identify which changes in environmental factors can benefit the mental health of older immigrant populations.

## Data Availability

The original contributions presented in the study are included in the article/supplementary material, further inquiries can be directed to the corresponding author.
